# Intergenerational and individual effects of smoking exposure on non-alcoholic fatty liver disease: A Mendelian randomization study

**DOI:** 10.18332/tid/226690

**Published:** 2026-09-24

**Authors:** Siqi Xie, Lan Liu, Bing Zhang, Jinna Zhang

**Affiliations:** 1 Fujian Children’s Hospital (Fujian Branch of Shanghai Children’s Medical Center), College of Clinical Medicine for Obstetrics and Gynecology and Pediatrics, Fujian Medical University, Fuzhou, Fujian, China; 2 Fujian Maternity and Child Health Hospital College of Clinical Medicine for Obstetrics and Gynecology and Pediatrics, Fujian Medical University, Fuzhou, Fujian, China

**Keywords:** maternal smoking around birth, early-life smoking, NAFLD, Mendelian randomization

## Abstract

**INTRODUCTION:**

Tobacco exposure has been linked to an increased risk of non-alcoholic fatty liver disease (NAFLD) in observational studies, yet evidence remains unclear due to confounding factors and reverse effect. This study aimed to explore the potential associations of maternal smoking around birth and age of smoking initiation with NAFLD risk.

**METHODS:**

Genome-wide association data for maternal smoking around birth, age of smoking initiation, and NAFLD were sourced from large-scale Genome-Wide Association Studies (GWAS) datasets. Qualified single nucleotide polymorphisms (SNPs) associated with the two exposures served as instrumental variables in two-sample Mendelian randomization analyses, which utilized the inverse-variance weighted (IVW) method, weighted median estimator, and MR-Egger regression to explore their potential associations with NAFLD. Sensitivity analyses confirmed no obvious directional pleiotropy, while significant heterogeneity was detected among included SNPs.

**RESULTS:**

Genetically predicted maternal smoking significantly elevated NAFLD susceptibility (IVW: OR=1.98; 95% CI: 1.16–3.39), validated by weighted median. No definitive link was found between smoking initiation age and NAFLD. No evidence supported a potential effect of NAFLD on maternal smoking around birth in reverse MR.

**CONCLUSIONS:**

This study supports a potential association between maternal smoking around birth and NAFLD, while smoking initiation age lacks sufficient genetic evidence.

## INTRODUCTION

Smoking is a well-established independent risk factor for multiple adverse maternal and perinatal outcomes^1^. Maternal smoking during pregnancy has been associated with miscarriage, preterm birth, and sudden infant death syndrome^2^, while also increasing the risk of infertility in women^3^. Moreover, a recent Mendelian randomization (MR) study in Asian populations suggested that maternal smoking during pregnancy may elevate the risk of hepatocellular carcinoma (HCC) in offspring^4^.

Non-alcoholic fatty liver disease (NAFLD) is currently the most common chronic liver disease worldwide, affecting approximately 20–30% of the general population and up to 80% of individuals with obesity^5^. NAFLD encompasses a broad clinical spectrum ranging from simple steatosis to non-alcoholic steatohepatitis (NASH), which can further progress to fibrosis, cirrhosis, and HCC^6^. To date, no approved pharmacological therapy is available for NAFLD, and current management mainly relies on behavioral modification, including weight reduction, increased physical activity, and dietary intervention^7^. Accumulating epidemiological evidence indicates that tobacco smoking is positively associated with NAFLD risk^8,9^. Importantly, this association is not limited to conventional combustible cigarettes; a Korean study further demonstrated that dual use of e-cigarettes and combustible cigarettes was also significantly associated with NAFLD^10^.

However, observational studies are inherently susceptible to residual confounding, selection bias, and reverse effect, limiting their ability to establish potential associations^11^. Although randomized controlled trials are considered the gold standard for causal inference, they are ethically impractical in the context of smoking exposure. Mendelian randomization (MR), which uses genetic variants as instrumental variables (IVs), provides an alternative approach for causal inference. Because genetic variants are randomly allocated at conception according to Mendel’s laws of inheritance, MR analyses are less vulnerable to confounding and reverse causation^12^. Furthermore, the increasing availability of large-scale Genome-Wide Association Studies (GWAS) and meta-analyses has greatly enhanced the applicability of MR in epidemiological research^13^. Two-sample MR, in particular, utilizes summary-level GWAS data from separate but comparable populations to estimate the potential effect of an exposure on an outcome.

Age of smoking initiation was selected as an important individual smoking indicator because early smoking initiation is closely associated with long-term smoking duration, high smoking intensity and cumulative tobacco exposure^14^. It is also a key behavioral variable widely discussed in tobacco-related epidemiological studies, and its potential relationship with NAFLD remains controversial and requires rigorous verification.

This two-sample MR study innovatively explores both intergenerational and individual effects of tobacco exposure across the life course. Unlike prior observational research, this study evaluates associations between maternal perinatal smoking, age at first smoking, and NAFLD risk using genetic IVs.

## METHODS

### Data sources

The study was conducted in accordance with the core assumptions and reporting framework of Mendelian randomization (MR), following the STROBE-MR guidelines^15^ (Supplementary file Table 1). To systematically explore causal links, we applied a bidirectional two-sample Mendelian randomization design based on publicly available GWAS summary statistics. Summary-level GWAS data for maternal smoking around birth were obtained from the UK Biobank dataset (ukb-b-17685), released in 2018^16^. This dataset included 397732 participants and 9851867 single nucleotide polymorphisms (SNPs).

GWAS summary statistics for age at smoking initiation were retrieved from the IEU OpenGWAS database (ieu-b-24)^17^, comprising 341427 individuals of European ancestry and 11894779 SNPs.

Outcome data for non-alcoholic fatty liver disease (NAFLD) were obtained from the EMBL-EBI GWAS database (ebi-a-GCST90091033), based on a large European cohort published in 2021^18^. The dataset included 778614 participants and 6784388 SNPs (Table 1).

**Table 1:** Characteristics of the datasets, IEU OpenGWAS 2018, 2019 and 2021

*Exposure*	*Web source*	*Sample size*	*SNP size*	*First author*	*Consortium*	*Year*	*Population*
Maternal smoking around birth	UK Biobank (ukb-b-17685)	397732	9851867	B. Elsworth	MRC-IEU	2018	European
Age of smoking initiation	IEU GWAS database (ieu-b-24)	341427	11894779	M. Liu	GWAS	2019	European
**Outcome**							
Non-alcoholic fatty liver disease (NAFLD)	EMBL-EBI (ebi-a-GCST90091033)	778614	6784388	N. Ghodsian	NA	2021	European

SNP: single nucleotide polymorphism. MRC-IEU: Medical Research Council Integrative Epidemiology Unit. GWAS: Genome-Wide Association Study. EMBL-EBI: European Molecular Biology Laboratory–European Bioinformatics Institute. NA: not available.

### Instrumental variable selection

To ensure the validity and reliability of the MR analysis, IVs were selected based on several stringent criteria. Only SNPs that were significantly associated with the exposure at the genome-wide significance level (p<5×10^-8^) were included. SNPs with a minor allele frequency (MAF) <0.01 in the outcome dataset were excluded to avoid instability caused by rare variants. To minimize bias arising from linkage disequilibrium (LD), SNPs were further pruned using an LD threshold of r^2^<0.001 within a 10000 kb window. In addition, potential pleiotropic variants associated with confounding factors or outcomes, particularly those related to chronic liver disease and alcohol consumption, were identified and removed using the *FastTraitR* package (version1.0.1).

The proportion of variance explained by each SNP was calculated using the following formula^19^:

R^2^ = [2β^2^×EAF×(1-EAF)]/[2β^2^×EAF×(1-EAF) + 2SE^2^×N×EAF×(1-EAF)]

where β represents the estimated effect size of the SNP on the exposure, EAF is the effect allele frequency, SE is the standard error, and N is the sample size. The strength of the instrumental variables was assessed using the F statistic derived from the proportion of explained variance (R^2^) and the number of included instruments. Generally, an F statistic <10 was considered indicative of weak instrumental variables, which may introduce bias into the estimates^20^.

### Statistical analysis

MR analyses rely on three core assumptions to ensure valid causal inference^19^: 1) the selected SNPs must be strongly associated with the exposure (relevance assumption); 2) the SNPs must affect the outcome only through the exposure (exclusion restriction assumption); and 3) the SNPs are not associated with any confounding factors between exposure and outcome (exchangeability assumption). Two-sample MR analyses were performed using R software (version 4.3.2) with the *TwoSampleMR* package (version 0.6.29). Three complementary MR methods were applied, including inverse variance weighted (IVW) analysis^19-21^, weighted median estimation^22^, and MR-Egger regression^23^. Potential associations were expressed as odds ratios (ORs) with corresponding 95% confidence intervals (CIs). A p<0.05 was considered statistically significant.

### Reverse Mendelian randomization framework

Bidirectional reverse two-sample MR was conducted to assess reverse causation (NAFLD → maternal smoking around birth). We selected genome-wide significant SNPs of NAFLD using the same thresholds as forward MR, with IVW as the primary method and leave-one-out analysis for robustness.

### Sensitivity analyses

Multiple sensitivity analyses were conducted to evaluate the robustness of the MR findings: MR-Egger intercept test to assess directional pleiotropy; Cochran’s Q test to evaluate heterogeneity among SNPs; leave-one-out analysis to determine whether individual SNPs disproportionately influenced the overall estimates^24^.

## RESULTS

### Characteristics of included SNPs

Detailed characteristics of all datasets are summarized in Table 1. Detailed information for the selected SNPs, including effect alleles, allele frequencies, and association estimates for both exposure and outcome datasets, is provided in Table 2.

**Table 2:** Association analysis for maternal smoking around birth/Age of smoking initiation -increasing GWAS risk alleles with the NAFLD, IEU OpenGWAS 2018, 2019 and 2021 (N=397732 for maternal smoking around birth; N=341427 for age of smoking initiation and N=778614 for Nonalcoholic Fatty Liver Disease (NAFLD))

*CHR*	*Position*	*SNPs*	*EA*	*EAF*	*Maternal smoking around birth*			*NAFLD*		
					*β*	*SE*	*P*	*β*	*SE*	*P*
1	44097438	rs12405972	T	0.35	-0.01	0.00	6.39E-14	-0.01	0.35	0.02
2	164928199	rs35566160	G	0.27	0.01	0.00	4.49E-08	0.01	0.28	0.00
4	140939110	rs36072649	A	0.38	-0.01	0.00	1.13E-11	-0.01	0.38	0.00
5	50748173	rs4865667	T	0.39	-0.01	0.00	3.42E-08	0.01	0.39	0.16
6	26159356	rs2183947	A	0.22	-0.01	0.00	1.54E-10	-0.01	0.23	0.01
7	32315613	rs10226228	G	0.37	0.01	0.00	3.97E-12	0.01	0.37	0.00
7	114951541	rs62477310	C	0.49	-0.01	0.00	2.00E-08	-0.01	0.49	0.00
7	75038408	rs77497827	G	0.47	0.01	0.00	1.25E-08	0.01	0.47	0.00
8	93114414	rs7002049	C	0.78	0.01	0.00	1.44E-09	-0.01	0.79	0.02
9	14453010	rs1323341	G	0.78	-0.01	0.00	3.89E-08	-0.01	0.78	0.00
9	136468701	rs75596189	T	0.11	0.01	0.00	2.15E-13	0.00	0.11	0.87
10	104727304	rs7899608	T	0.14	0.01	0.00	2.34E-09	0.01	0.14	0.04
11	113678423	rs2428019	A	0.24	0.01	0.00	5.08E-09	-0.01	0.24	0.00
15	78870803	rs576982	T	0.23	-0.01	0.00	2.28E-14	-0.02	0.23	0.43
16	24798079	rs12923476	A	0.26	-0.01	0.00	4.82E-09	-0.02	0.26	0.01
20	61984317	rs6011779	T	0.81	-0.01	0.00	2.50E-14	0.00	0.81	0.13
***CHR***	***Position***	***SNPs***	***EA***	***EAF***	***Age of Smoking Initiation***			***NAFLD***		
					***β***	***SE***	***P***	***β***	***SE***	***P***
2	63613544	rs10200107	A	0.56	-0.02	0.00	1.89E-12	0.00	0.00	0.21
2	225450161	rs3768886	C	0.33	0.02	0.00	7.50E-09	0.00	0.00	0.13
3	85699040	rs11915747	G	0.35	0.02	0.00	3.89E-13	0.01	0.00	0.11
4	2881256	rs624833	G	0.31	0.02	0.00	8.75E-09	-0.01	0.00	0.05
8	27344719	rs11780471	A	0.06	0.04	0.01	7.07E-11	-0.02	0.01	0.01
15	75360268	rs140485736	A	0.01	0.07	0.01	1.39E-08	0.01	0.01	0.60
17	31554533	rs319748	A	0.71	-0.02	0.00	3.01E-08	-0.01	0.00	0.03

N=397732 for maternal smoking around birth; N=341427 for age of smoking initiation; and N=778614 for non-alcoholic fatty liver disease (NAFLD). Statistical significance was defined as p<0.05 for MR analyses. Genome-wide significance for SNP selection was set at p<5×10^-8^. CHR: chromosome. EA: effect allele. EAF: effect allele frequency. SE: standard error. SNP: single-nucleotide polymorphism.

### Potential effects of smoking-related exposures on NAFLD

**Figure 1:**
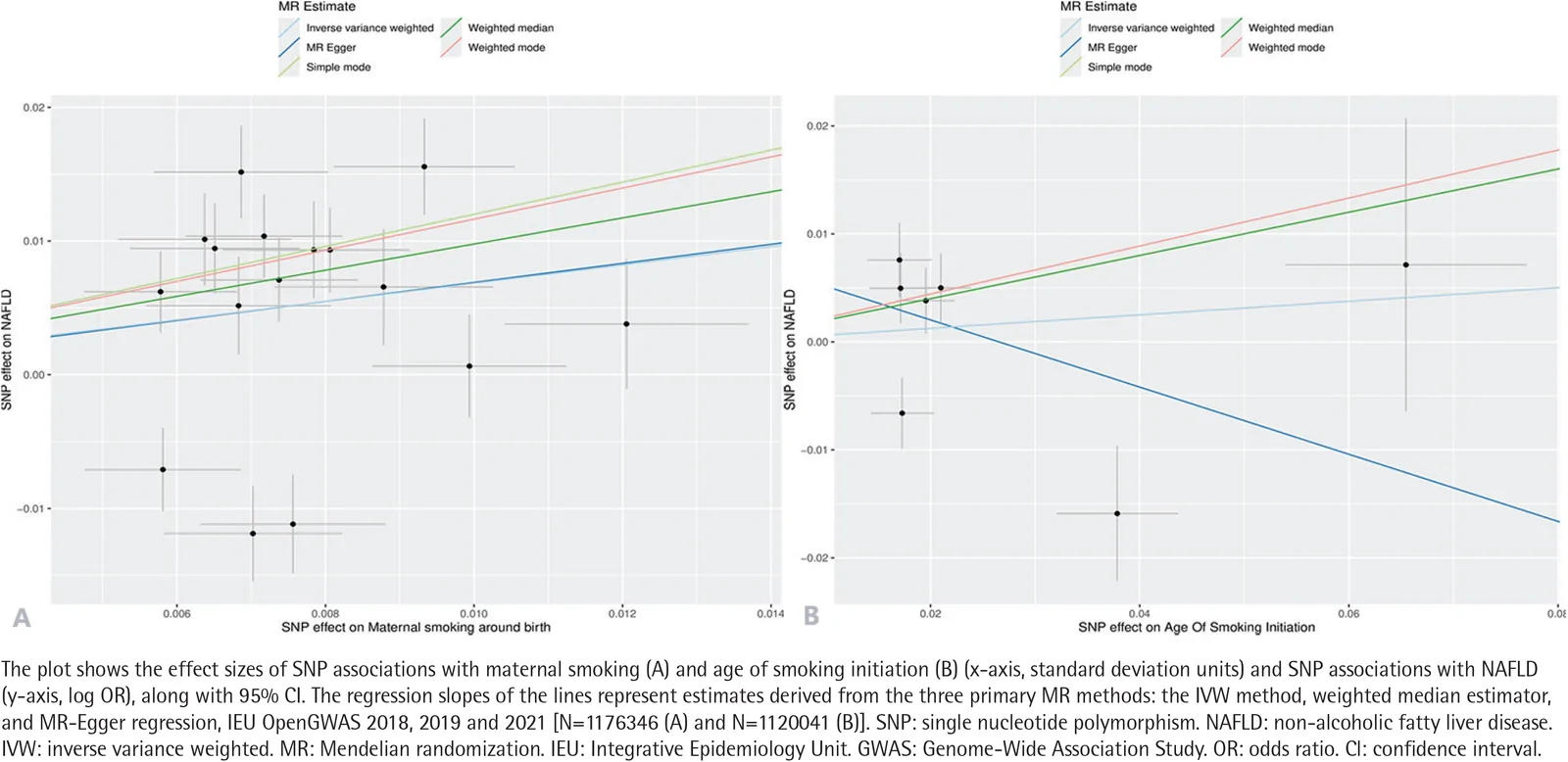
Scatter plot of the SNPs associated with maternal smoking around birth (A), age of smoking initiation (B), and the risk of NAFLD

**Figure 2:**
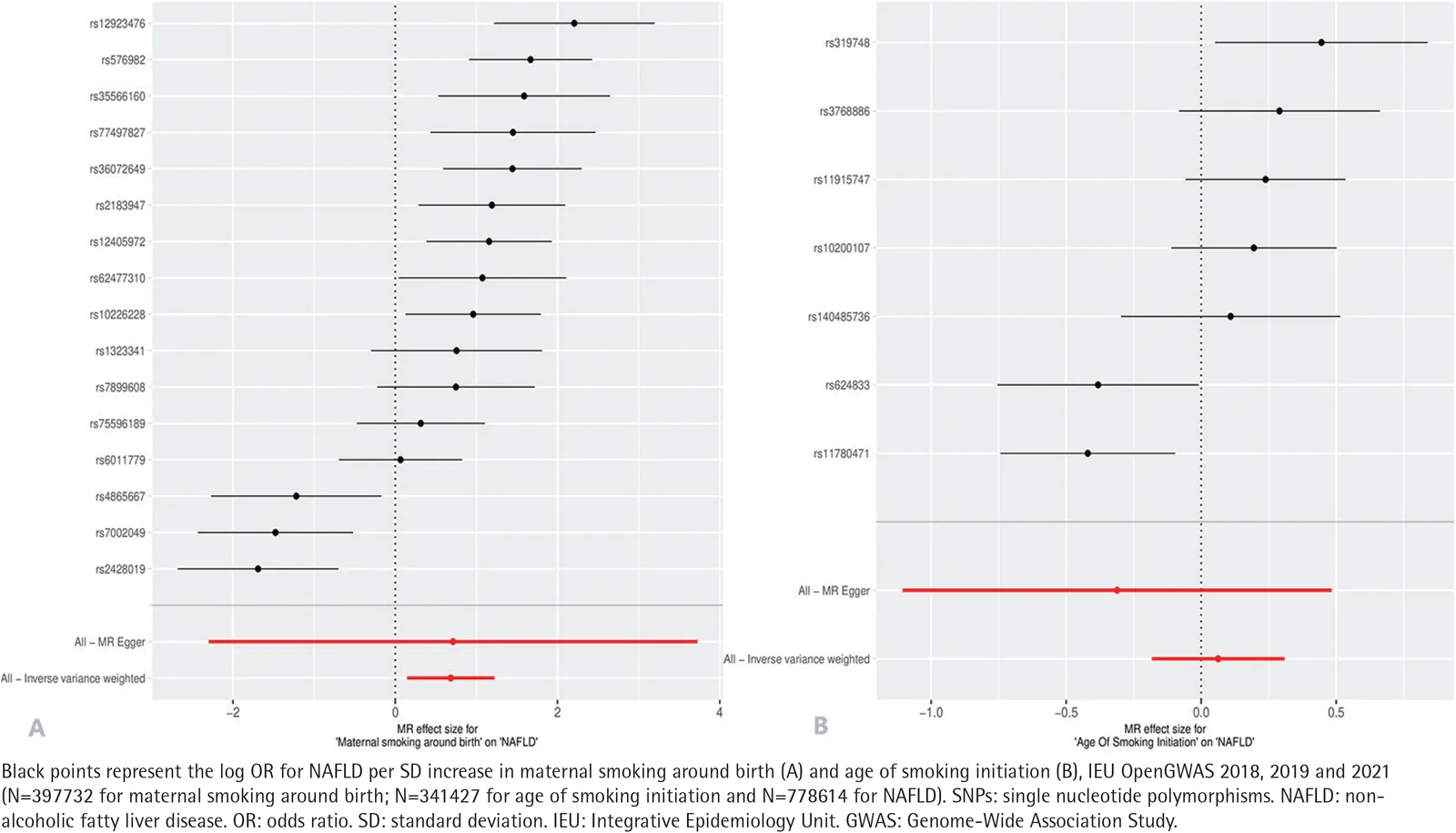
Forest plot of SNPs associated with maternal smoking around birth (A), age of smoking initiation (B), and the risk of NAFLD

For maternal smoking around birth, IVW analysis demonstrated a significant positive association with NAFLD risk (OR=1.98; 95% CI: 1.16–3.39, p=0.01) (Table 3). This finding was further supported by the weighted median analysis (OR=2.66; 95% CI: 1.80–3.93, p=0.00). Although the MR-Egger estimate showed a similar direction of effect, the association did not reach statistical significance (OR=2.04; 95% CI: 0.10–41.46, p=0.65) (Figure 1A). Forest plots demonstrated overall consistency across analytical methods (Figure 2A).

**Table 3:** Mendelian randomization results for the association between the two exposures and the outcome NAFLD, IEU OpenGWAS 2018, 2019 and 2021

*Exposure*	*Forward MR*	*β*	*SE*	*OR (95% CI)*	*p*
**Maternal smoking around birth**	MR Egger	0.71	1.54	2.04 (0.10–41.46)	0.65
	Weighted median	0.98	0.20	2.66 (1.80–3.93)	0.00
	IVW	0.68	0.27	1.98 (1.16–3.39)	0.01
	Simple mode	1.20	0.26	3.32 (1.98–5.56)	0.00
	Weighted mode	1.16	0.30	3.20 (1.77–5.79)	0.00
**Age of smoking initiation** (years)	MR Egger	-0.31	0.41	0.73 (0.33–1.62)	0.48
	Weighted median	0.20	0.10	1.22 (1.01–1.48)	0.04
	IVW	0.06	0.13	1.07 (0.83–1.36)	0.62
	Simple mode	0.22	0.12	1.25 (0.98–1.59)	0.13
	Weighted mode	0.22	0.11	1.25 (1.01–1.55)	0.09

N=397732 for maternal smoking around birth; N=341427 for age of smoking initiation; and N=778614 for non-alcoholic fatty liver disease (NAFLD). Statistical significance was defined as p<0.05 for MR analyses; Genome-wide significance for SNP selection was set at p<5×10^-8^. All effect estimates for age at smoking initiation correspond to a one-year increment in the age of first smoking, and no separate reference category was defined for this exposure. MR: Mendelian randomization. IVW: Inverse variance weighted. OR: odds ratio. CI: confidence interval. SE: standard error. SNP: single-nucleotide polymorphism.

For age of smoking initiation, where each effect estimate corresponds to a one-year increase in the age at first smoking with no designated reference group, IVW analysis did not reveal a significant association with NAFLD risk (OR=1.07; 95% CI: 0.83–1.36, p=0.62) (Table 3). Similar null findings were observed in MR-Egger regression (OR=0.73; 95% CI: 0.33–1.62, p=0.48). Although the weighted median method showed a nominally significant association (OR=1.22; 95% CI: 1.01–1.48, p=0.04), the overall evidence was insufficient to support a robust potential relationship (Figure 1B). Forest plots indicated general consistency among methods (Figure 2B).

### Sensitivity analyses

**Figure 3:**
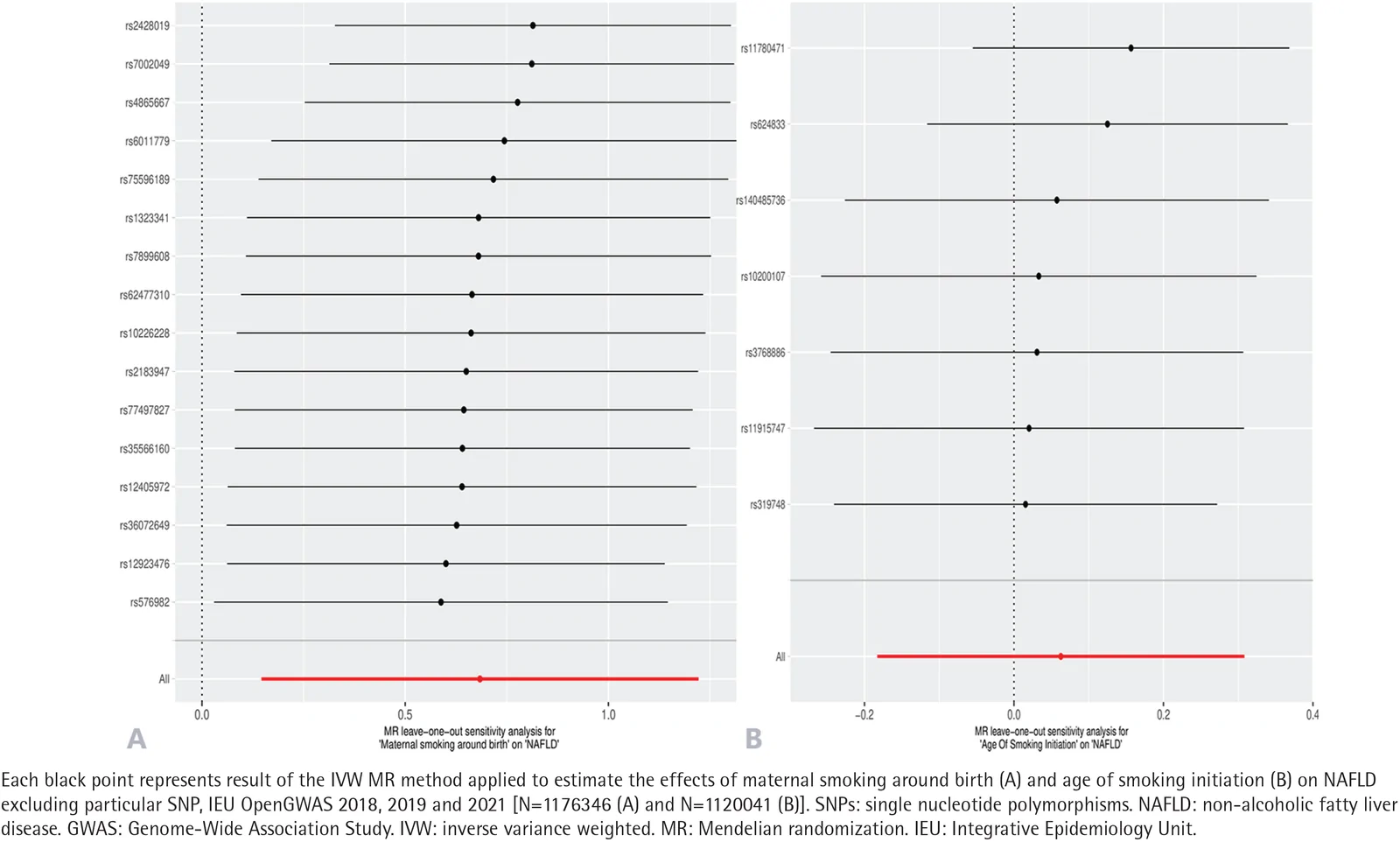
Leave-one-out of SNPs associated with maternal smoking around birth (A), age of smoking initiation (B), and the risk of NAFLD

The MR-Egger intercept test showed no evidence of directional pleiotropy (p=0.985). However, Cochran’s Q test indicated significant heterogeneity among the included SNPs (Table 4). Leave-one-out analyses demonstrated that no single SNP substantially influenced the overall estimates for either maternal smoking (Figure 3A) or age at smoking initiation (Figure 3B), supporting the robustness of the primary findings.

**Table 4:** Heterogeneity and pleiotropy tests, IEU OpenGWAS 2018, 2019 and 2021

*Exposure*	*Outcome*	*Heterogeneity test*				*Pleiotropy test*
		*IVW Q*	*p*	*MR-Egger Q*	*p*	*MR-Egger p*
ukb-b-17685	ebi-a-GCST90091033	84.97	0.00	84.97	0.00	0.99
ieu-b-24	ebi-a-GCST90091033	21.20	0.00	17.84	0.00	0.38

N=397732 for maternal smoking around birth; N=341427 for age of smoking initiation; and N=778614 for non-alcoholic fatty liver disease (NAFLD). Statistical significance was defined as p<0.05 for MR analyses; Genome-wide significance for SNP selection was set at p<5×10^-8^. IVW: inverse variance weighted. Q: Cochran’s Q statistic. MR: Mendelian randomization. SNP: single-nucleotide polymorphism.

### Reverse Mendelian randomization analysis

**Figure 4:**
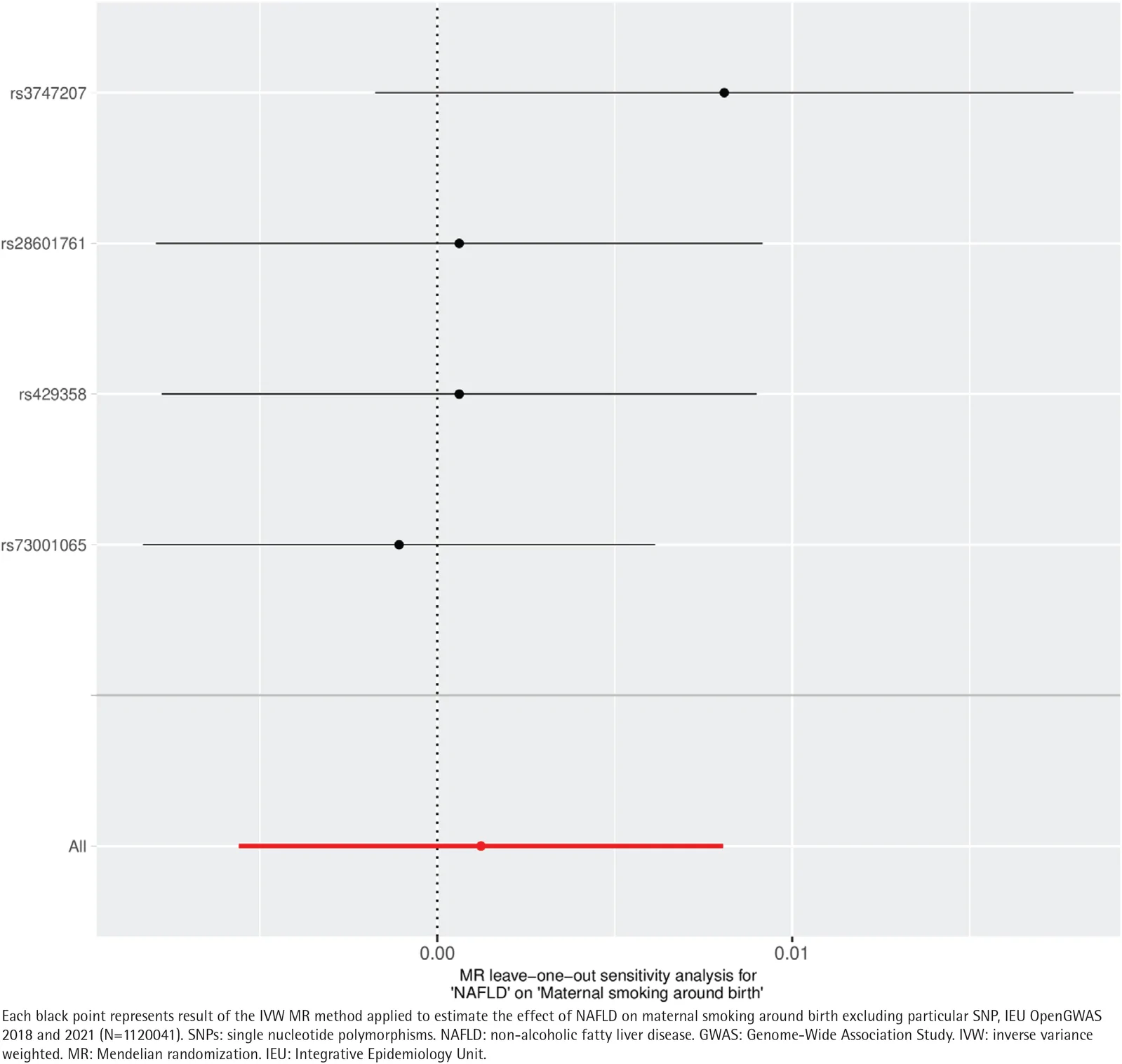
Leave-one-out of SNPs associated with NAFLD and the risk of maternal smoking around birth

Reverse MR analysis was performed using four independent SNPs significantly associated with NAFLD (p<5×10^-8^). IVW analysis found no evidence of a potential effect of NAFLD on maternal smoking around birth (Supplementary file Table 2), further supporting the directionality of the primary MR findings. Although some heterogeneity was observed in the leave-one-out analyses, the overall estimates remained stable (Figure 4).

## DISCUSSION

This MR analysis found that genetic proxies for maternal smoking around birth were linked to elevated NAFLD risk, while no robust genetic association was observed between age of smoking initiation and NAFLD. These findings partially align with previous observational studies that reported a positive association between maternal smoking and offspring liver diseases. A prior MR study also indicated that maternal smoking was associated with higher risk of hepatocellular carcinoma in offspring, further supporting the adverse intergenerational effects of perinatal tobacco exposure^4,25^. Multiple studies have revealed the biological links between smoking and liver metabolism. Nicotine can alter intestinal molecular signaling and gut microbiota composition, further accelerating the progression of NAFLD^26-28^. Perinatal tobacco exposure is common, and it may disturb fetal metabolic status and early gut microbial homeostasis^29,30^.

In the present MR study, this study systematically investigated the genetic potential relationships between maternal smoking around birth, age at smoking initiation, and NAFLD risk using well-characterized SNPs derived from large-scale genome-wide association study datasets. Consistent with the primary hypothesis, multiple MR approaches supported a robust positive effect of genetically predicted maternal smoking exposure on NAFLD susceptibility. From the perspective of the Developmental Origins of Health and Disease (DOHaD), intrauterine exposure to tobacco smoke may impair fetal metabolic programming^31^, disrupt hepatic lipid homeostasis, trigger persistent inflammatory activation, and promote ectopic lipid accumulation in the liver^32,33^, ultimately increasing long-term susceptibility to fatty liver disease in offspring. In contrast, no definitive potential association was observed between age at smoking initiation and NAFLD risk in the primary IVW analysis. The discrepancy between MR findings and previous observational studies may be explained by residual confounding from behavioral factors, socioeconomic status, smoking intensity, and other behavioral variables, rather than a direct biological effect.

Although Cochran’s Q test indicated significant heterogeneity among genetic instruments, leave-one-out sensitivity analyses confirmed that no single SNP disproportionately influenced the overall estimates. These findings suggest that the results were not driven by individual outlier variants and remain relatively robust despite underlying heterogeneity.

Importantly, bidirectional MR analysis did not support a reverse effect of NAFLD on maternal smoking around birth, thereby reinforcing a unidirectional relationship in which maternal smoking precedes NAFLD development. This further reduces concerns regarding reverse causation bias, which is common in conventional observational studies.

### Limitations

Several limitations should be acknowledged. First, the analysis was restricted to individuals of European ancestry, limiting generalizability. Second, the relatively limited number of valid instruments may reduce statistical power and increase the risk of false-negative findings. Third, subgroup analyses stratified by sex, age, or smoking intensity were not performed. Additionally, the limited number of valid genetic instruments may introduce weak instrument bias, which could affect the accuracy of causal estimates and increase the possibility of false-negative results. Finally, the biological mechanisms linking maternal smoking to NAFLD remain incompletely understood and require further experimental validation, particularly regarding epigenetic regulation, inflammatory signaling, and lipid metabolism pathways.

## CONCLUSIONS

This Mendelian randomization study provides genetic evidence that maternal smoking around birth supports a potential risk factor for increased NAFLD risk, whereas age at smoking initiation shows no convincing genetic potential association with NAFLD. These findings offer new insights into the developmental origins of NAFLD. Future research is needed to validate these findings in non-European populations, explore sex-specific subgroups, and clarify the epigenetic and molecular mechanisms underlying the association between maternal smoking and NAFLD.

## Supplementary Material



## Data Availability

The data supporting this research are available from the authors on reasonable request.
